# High nuclear TPX2 expression correlates with TP53 mutation and poor clinical behavior in a large breast cancer cohort, but is not an independent predictor of chromosomal instability

**DOI:** 10.1186/s12885-021-07893-7

**Published:** 2021-02-23

**Authors:** Daniel R. Matson, Ryan A. Denu, Lauren M. Zasadil, Mark E. Burkard, Beth A. Weaver, Christopher Flynn, P. Todd Stukenberg

**Affiliations:** 1 Department of Pathology and Laboratory Medicine, University of Wisconsin-Madison, 600 Highland Ave, Madison, WI 53792 USA; 2 Department of Medicine, University of Wisconsin Hospitals and Clinics, Madison, WI USA; 3 Molecular and Cellular Pharmacology Training Program, University of Wisconsin-Madison, Madison, WI USA; 4 Carbone Cancer Center, University of Wisconsin-Madison, Madison, WI USA; 5 Department of Oncology/McArdle Laboratory for Cancer Research, University of Wisconsin-Madison, Madison, WI USA; 6 Department of Cell and Regenerative Biology, University of Wisconsin-Madison, Madison, WI USA; 7 Department of Biochemistry and Molecular Genetics, University of Virginia, Charlottesville, VA USA

**Keywords:** MeSH: breast neoplasms, Chromosomal instability, Tumor suppressor protein p53, Pathology, Other: Targeting Protein for Xenopus Kinesin Like Protein 2 (TPX2).

## Abstract

**Background:**

Targeting Protein for Xenopus Kinesin Like Protein 2 (TPX2) is a microtubule associated protein that functions in mitotic spindle assembly. TPX2 also localizes to the nucleus where it functions in DNA damage repair during S-phase. We and others have previously shown that TPX2 RNA levels are strongly associated with chromosomal instability (CIN) in breast and other cancers, and TPX2 RNA levels have been demonstrated to correlate with aggressive behavior and poor clinical outcome across a range of solid malignancies, including breast cancer.

**Methods:**

We perform TPX2 IHC on a cohort of 253 primary breast cancers and adopt a clinically amenable scoring system to separate tumors into low, intermediate, or high TPX2 expression. We then correlate TPX2 expression against diverse pathologic parameters and important measures of clinical outcome, including disease-specific and overall survival. We link TPX2 expression to TP53 mutation and evaluate whether TPX2 is an independent predictor of chromosomal instability (CIN).

**Results:**

We find that TPX2 nuclear expression strongly correlates with high grade morphology, elevated clinical stage, negative ER and PR status, and both disease-specific and overall survival. We also show that increased TPX2 nuclear expression correlates with elevated ploidy, supernumerary centrosomes, and TP53 mutation. TPX2 nuclear expression correlates with CIN via univariate analyses but is not independently predictive when compared to ploidy, Ki67, TP53 mutational status, centrosome number, and patient age.

**Conclusions:**

Our findings demonstrate a strong correlation between TPX2 nuclear expression and aggressive tumor behavior, and show that TPX2 overexpression frequently occurs in the setting of TP53 mutation and elevated ploidy. However, TPX2 expression is not an independent predictor of CIN where it fails to outperform existing clinical and pathologic metrics.

**Supplementary Information:**

The online version contains supplementary material available at 10.1186/s12885-021-07893-7.

## Background

Targeting Protein for Xenopus Kinesin Like Protein 2 (TPX2) is a microtubule associated protein which critically regulates the formation of the mitotic spindle during mitosis, primarily by localizing and activating Aurora A kinase [1]. TPX2 also enhances microtubule nucleation independent of Aurora A, assists in the targeting of mitotic kinesins to microtubule minus ends, and works in concert with Augmin to form microtubule branch points [2]. Together, these activities maintain genomic stability by ensuring the proper segregation of chromosomes during mitosis. During interphase, TPX2 localizes to the nucleus where it plays a role in the DNA damage response. The TPX2-Aurora A heterodimer binds and counteracts Tumor Protein P53 Binding Protein 1 (53BP1) activity to stabilize and protect stalled DNA replication forks that occur in the setting of DNA damage [3]. It does this at least in part through recruitment of Breast Cancer 1 (BRCA1) and RAD51 Recombinase (RAD51). TPX2 depletion results in an accumulation of cells in mitosis and it is required for the synthesis and phosphorylation of p53 in *Xenopus* oocytes [4–7].

TPX2 is broadly implicated in oncogenesis in diverse solid organ malignancies, where increased levels of TPX2 mRNA typically correlates with unfavorable prognosis [8–12]. A large network analysis of gene expression profiles derived from two large human breast cancer cohorts and multiple mouse models of metastatic disease identified a conserved genetic signature involving TPX2 which was associated with distant metastases and worse survival [13]. TPX2 has been strongly implicated in the survival of genomically unstable cancers and targeting of TPX2 in cancer cell lines leads to mitotic arrest and increased genomic instability [14, 15]. In breast cancers, TPX2 mRNA levels have been reported as a strong predictor of aggressive behavior, reduced response to therapy, and poor survival, while depletion of TPX2 can suppress proliferation and promote apoptosis [16–22]. However, to our knowledge, a large immunohistochemistry (IHC)-based study of TPX2 protein expression has not been performed in primary breast cancers.

Chromosomal instability (CIN) occurs when cells randomly gain or lose whole chromosomes or large segments of chromosomes during mitosis, and along with microsatellite instability is one of the primary forms of genomic instability found in cancer [23, 24]. The presence of CIN strongly correlates with poor prognosis in solid tumors and is linked to the acquisition of resistance to chemotherapy [25, 26]. Unfortunately, measuring CIN in patient tumors requires the manual scoring of multi-centromere fluorescence in situ hybridization (FISH) across at least 100 tumor cells, a laborious process which is challenging to implement in routine clinical practice. In an effort to develop tractable methods to measure CIN in the clinic, we and others have utilized publicly available RNAseq datasets generated from patient-derived tumors to identify individual genes whose expression is linked to CIN [25, 27]. Out of all measured transcripts, expression of the gene TPX2 was found to be the most highly correlated to CIN.

Here we perform TPX2 IHC on a cohort of 253 patient-derived breast cancers and correlate these findings with available clinical, pathological, and molecular findings. We find that increased TPX2 nuclear expression is significantly associated with tumor grade, clinical stage, estrogen receptor (ER) status, progesterone receptor (PR) status, and both disease-specific and overall survival. Furthermore, we evaluate the relationship between TPX2 nuclear expression and genomic instability by comparing TPX2 nuclear expression levels to CIN, ploidy, centrosome number, and TP53 mutational status. We find that increased TPX2 nuclear expression is significantly associated with higher average ploidy, increased rates of centrosome amplification, and a greater incidence of TP53 mutation. However, while TPX2 expression correlates with CIN in univariate analyses, it is not independently predictive of CIN when analyzed alongside available clinical and pathologic markers. Finally, we show that increased TPX2 nuclear expression strongly correlates with survival, but its predictive power in this setting is not independent of Ki67. Together, our results demonstrate the strong correlation between TPX2 expression and both molecular and clinical metrics of aggressive disease and poor clinical outcome, while also arguing that TPX2 IHC is not an appropriate standalone assay for the determination of CIN in human tumor samples.

## Methods

### Tissue microarray and IRB approval

The breast cancer tissue microarray (TMA) used in this study has been reported previously [28–30]. Briefly, the samples were obtained from primary Stage I-III breast tumor blocks obtained at the time of surgery from patients being treated at University of Wisconsin Carbone Cancer Center under protocol OS10111. Creation of the de-identified TMA and clinical data set were approved by the University of Wisconsin Health Sciences Institutional Review Board (IRB approval 2010–0405). The IRB waived patient consent. All cases contain at least 5 years of clinical follow-up or documented death or recurrence within 5 years. Linked clinical data included age at diagnosis, ethnicity, tumor size, lymph node involvement, stage, Ki67 index, estrogen receptor (ER), progesterone receptor (PR), and HER2 status, type of surgery, adjuvant breast cancer treatments, and follow-up data including recurrence and death. In addition, immunohistochemistry for Ki67 had been performed using a clinically validated antibody and automated scoring was performed using Vectra software. A total of 253 formalin-fixed paraffin-embedded patient-derived breast cancers with triplicate 0.6 mm punches were evaluated for the purposes of this study. These were derived from a total of 324 patient-derived breast cancers. After evaluation by a pathologist, 30 of these cases were determined to represent sampling of in situ disease and were not analyzed. An additional 41 cases could not be evaluated due to tissue loss during processing.

### Immunohistochemistry and scoring

Unstained 5 μm-thick sections were stained with anti-TPX2 (Millipore-Sigma, St. Louis, MO, USA) and anti-p53 (Clone DO-7, Roche, Basel, Switzerland) antibodies using the Ventana Discovery XT BioMarker Platform (Ventana, Tucson, AZ, USA). The anti-p53 antibody is clinically validated, while the anti-TPX2 antibody has been utilized in diverse tissue types [31–34]. For the anti-TPX2 antibody, deparaffinization and heat-based epitope retrieval were carried out on the instrument with buffer CC1 (Ventana #950–500) for 60 min at 100 °C. Anti-TPX2 was diluted 1:100 in Renaissance Background Reducing Agent diluent (BioCare Medical #PD905H) and incubated for 60 min at 37 °C. Sections were rinsed with Reaction Buffer (Ventana #950–300) and then incubated with Discovery OmniMap anti-Rabbit HRP (Ventana #760–4311) for 16 min at 37 °C. Sections were rinsed with Reaction Buffer and developed with Discovery ChromoMap DAB detection (Ventana #760–159), then counterstained with hematoxylin. Staining with the anti-p53 antibody was performed in a similar manner with the following exceptions: epitope retrieval was performed for 44 min, primary antibody incubation was performed for 28 min, and UltraMap anti-Ms HRP was applied for 8 min.

TPX2 and p53 stained TMAs were scored by a pathologist (DRM) in a blinded manner. All available tissues cores were scored for each tumor and the average score across all cores was used for analyses. For TPX2, the percent of malignant cells with positive nuclear or cytoplasmic staining as well as the intensity of nuclear and cytoplasmic staining were scored. Intensity was scored on a scale from 0 to 3: 0 = completely negative, 1 = blush or dim, 2 = moderate diffuse or heterogeneous, 3 = strong and diffuse. For p53, the percent of positive nuclei were scored. Tumors were considered to have an aberrant p53 IHC pattern if the percent of p53-positive cells was < 1% or greater than 90%. This yielded a 25% rate of TP53 mutation, which is consistent with previously reported rates of TP53 mutation in large cohorts of primary breast tumors [35]. Centrosome number had been previously quantified using anti-pericentrin (Abcam, Cambridge, UK) and anti-polyglutamylated tubulin (Adipogen, San Diego, CA, USA) antibodies [29]. Centrosomes were quantified in at least 30 cells per tumor by blinded observers.

### Chromosome instability

Measurements of chromosome instability (CIN) had been performed previously [29]. Briefly, chromosomes 3, 4, 7, 9, 10, and 17 were probed by FISH and chromosome numbers per cell were quantified by blinded observers in at least 10 cells per tumor. Ploidy was determined by the average combined number of all probes per cell. CIN was calculated as the average number of cells that deviated from the modal number for each analyzed chromosome. Samples were considered to have CIN if this value exceeded 45%, which yielded an appropriate ratio of CIN to non-CIN tumors.

### Statistical methods

Statistics and survival analyses were performed in GraphPad PRISM version 8 (GraphPad Software Inc., La Jolla, CA, USA) with multivariate analyses performed in R v4.0.3 (The R Foundation for Statistical Computing). Comparisons of TPX2 nuclear expression with age, tumor grade, clinical stage, histology, receptor status, and regional lymph node expression were analyzed using *X*^2^. When appropriate, post-hoc testing of *X*^2^ tests was performed via partitioning and Fisher’s exact test. Comparisons of nuclear TPX2 expression with CIN, mutated TP53, ploidy, and centrosome number were performed using ANOVA and post-hoc testing was performed using Tukey’s multiple comparisons testing. Overall survival was plotted using the Kaplan-Meier method. Statistical significance of disease-specific survival and overall survival were analyzed using log-rank (Mantel-Cox) testing.

## Results

### Distribution of TPX2 across human breast cancers

Utilizing a publicly available cohort of 105 primary human breast cancers within The Cancer Genome Atlas for which both genome-wide RNAseq and quantitative proteomics data were available, we confirmed the significant and previously-reported positive correlation between TPX2 RNA expression and TPX2 protein levels (Supplemental Figure 1A) [36, 37]. IHC for TPX2 was then performed on three TMA slides collectively representing 253 patient-derived primary breast cancers with linked pathological and clinical parameters (Table 1). All patients were female with a median age of 55 years. The majority of tumors had ductal histology (81%) with a smaller subset of lobular carcinomas (8%) and a mixture of less common histologic subtypes. Tumors represented a mix of Grade 1 (21%), Grade 2 (43%), and Grade 3 (36%) histology. Most tumors were ER-positive (80%) and PR-positive (71%), and a small subset were HER2-positive (17%). All clinical stages were represented with most tumors derived from Stage 1 (40%) or Stage 2 (48%) disease and a smaller number of Stage 3 (12%) cases. Lymph node metastases were present at diagnosis in 41% of patients. Recurrences occurred in 22% of patients and 15% of patients died due to breast cancer.

**Table 1:** Clinical and pathological parameters of breast cancer cohort

Parameter	Number	%
**Number of patients**	253	
**Age, median (range)**	55	(27–94)
**Sex**		
Female	253	100
Male	0	0
**Histology**		
Ductal	204	81
Lobular	20	8
Ductal and lobular	9	4
Carcinoma NOS	3	1
Mucinous	3	1
Ductal mixed with other	2	1
Lobular mixed with other	2	1
Acinar	1	< 1
Intracystic	1	< 1
Malignant phyllodes	1	< 1
Metaplastic	1	< 1
**Tumor Grade**		
1	52	21
2	107	43
3	91	36
**Receptor Status**		
ER+	198	80
PR+	177	71
HER2+	32	17
**Clinical Stage**		
1	102	40
2	9	4
2A	73	29
2B	38	15
3	2	1
3A	16	6
3B	7	3
3C	5	2
**Regional Lymph Nodes**		
Positive	103	41
Negative	150	59
**Death Due to Breast Cancer**		
Yes	39	15
No	214	85

**Fig. 1:**
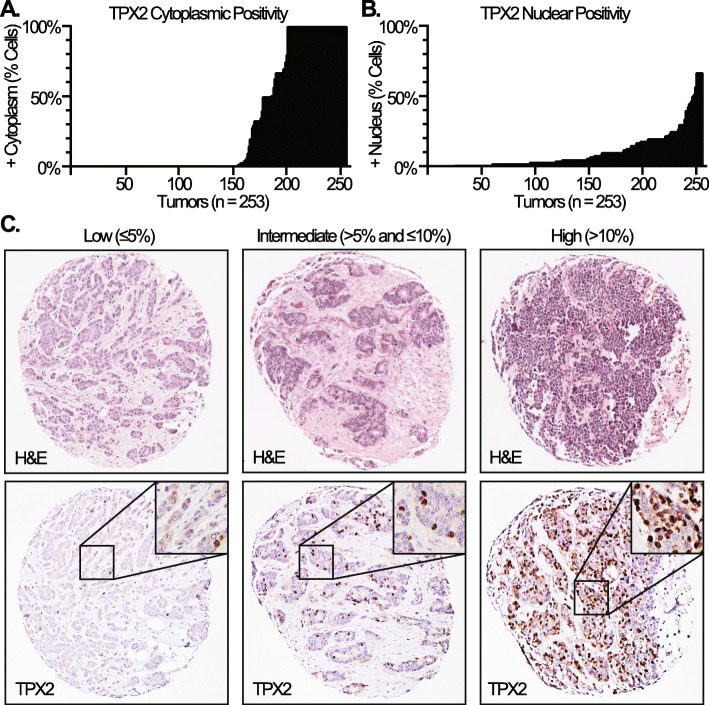
**a** Histogram depicting percent of cells with TPX2 cytoplasmic expression in all scored tumors. **b**, Histogram depicting percent of all cells with TPX2 nuclear expression in all scored tumors. **c**, Representative images depicting H&E and TPX2 IHC from tumors with low, intermediate, and high TPX2 nuclear expression (magnification: 100X, insets 400X)

The percentage of cells with positive TPX2 staining in the cytoplasm and/or nucleus were quantified across all tumors. The average intensity of TPX2 staining was also scored using a 4-point scale (0-negative, 1-low, 2-intermediate, 3-high). We found that the intensity of TPX2 staining was strong in virtually all tumors that demonstrated any level of TPX2 expression, and that TPX2 staining intensity did not correlate with any clinicopathologic parameters (data not shown). The percentage of cells with cytoplasmic TPX2 expression was largely bimodal, with most tumors showing either a complete lack of cytoplasmic TPX2 or essentially all cells demonstrating cytoplasmic TPX2 staining, although approximately 20% of tumors displayed only partial cytoplasmic TPX2 expression (Fig. 1a). The percentage of cells with TPX2 nuclear expression showed a more nuanced distribution which ranged from 0 to 67% (Fig. 1b). We found no significant correlation between cytoplasmic and nuclear TPX2 expression (Pearson r = − 0.12 and *P* = 0.065). We next stratified the TPX2 nuclear expression data into categories which would be more amenable to pathological scoring in routine surgical pathology practice and meaningfully separate the tumors into groups for comparison. To this end, we chose levels of low (≤5%), intermediate (> 5% and ≤ 10%), and high (> 10%) which were utilized for all statistical analyses (Fig. 1c).

### TPX2 nuclear expression and Clinicopathologic parameters

We first sought to determine whether there were significant differences in clinicopathologic parameters across tumors with low, intermediate, and high TPX2 nuclear expression. We found that increased TPX2 nuclear expression showed a significant correlation with higher tumor grade, higher clinical stage, negative ER status, and negative PR status (Table 2). TPX2 nuclear expression showed no significant correlation with patient age at diagnosis, tumor histology, HER2 receptor status, or the presence of lymph node metastases at the time of diagnosis. In contrast to nuclear TPX2, cytoplasmic TPX2 expression did not correlate with any of the analyzed clinicopathologic parameters.

**Table 2:** Statistical relationships between TPX2 nuclear expression and age, tumor grade, clinical stage, tumor histology, receptor status, and presence or absence of lymph node metastases at the time of diagnosis. Percentages are rounded to the nearest whole number and may not sum to 100%

	TPX2 Nuclear Expression			
**Parameters**	**Low** **(*n*** **= 150)** **n (%)**	**Intermediate** **(*n*** **= 34)** **n (%)**	**High** **(*n*** **= 69)** **n (%)**	χ**2 (*P*-value**)
**Age**				
≤ 50 years	59 (39)	12 (35)	31 (45)	1.0 (0.599)
> 50 years	91 (61)	22 (65)	38 (55)	
**Tumor Grade**				**75.6 (< 0.0001)**
1	47 (32)	2 (6)	3 (4)	
2	77 (52)	16 (48)	14 (21)	
3	25 (17)	15 (45)	51 (75)	
**Clinical Stage**				**23.5 (< 0.0001)**
1	79 (53)	9 (27)	14 (20)	
2	56 (37)	20 (61)	44 (64)	
3	15 (10)	4 (12)	11 (16)	
**Histology**				2.9 (0.579)
Ductal	119 (79)	26 (76)	59 (86)	
Lobular	13 (9)	2 (6)	5 (7)	
Other	18 (12)	6 (18)	5 (7)	
**Receptor Status**				
ER**+**	141 (95)	24 (73)	33 (49)	**59.5 (< 0.0001)**
ER**-**	8 (5)	9 (27)	34 (51)	
PR**+**	122 (82)	20 (61)	35 (52)	**22.7 (< 0.0001)**
PR**-**	26 (18)	13 (39)	32 (48)	
HER2**+**	17 (16)	6 (22)	9 (19)	0.7 (0.689)
HER2**-**	92 (84)	21 (78)	39 (81)	
**Regional Lymph Nodes**				
Positive	56 (37)	15 (44)	32 (46)	1.8 (0.409)
Negative	94 (63)	19 (56)	37 (54)	

### TPX2 nuclear expression and tumor proliferation

**Fig. 2:**
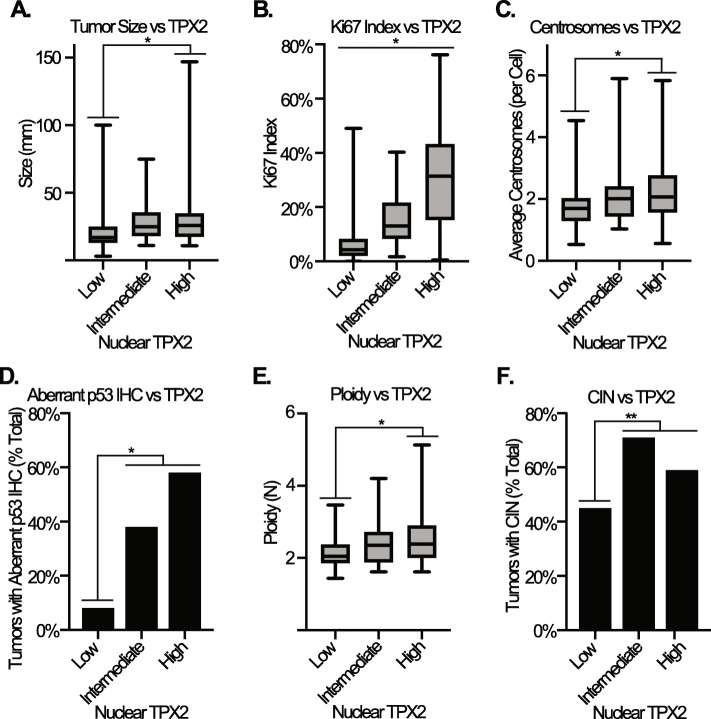
**a** Tumor size versus TPX2 nuclear expression. **b**, Ki67 index versus TPX2 nuclear expression. **c**, Average number of centrosomes per cell versus TPX2 nuclear expression. **d**, Percent of tumors with aberrant p53 immunohistochemical staining versus TPX2 nuclear expression. **e**, Average ploidy versus TPX2 nuclear expression. **f**, Percentage of tumors with CIN versus TPX2 nuclear expression. *P < 0.0001, **P < 0.005

Under normal biological conditions, TPX2 plays at least two cell-cycle specific roles to ensure timely cell proliferation: 1) it regulates mitotic spindle assembly and 2) it regulates the response to DNA damage during S-phase. Therefore, we sought to determine if increased expression of TPX2 correlated with markers of cell proliferation, including tumor size and Ki67 index. We found that TPX2 nuclear expression correlated with an increase in tumor size, with larger tumors demonstrating higher TPX2 nuclear expression (Fig. 2a). TPX2 nuclear expression also showed a striking correlation with increased Ki67 index which was statistically significant across all groups (Fig. 2b). Follow up linear regression analysis confirmed a highly significant, positive correlation between TPX2 nuclear expression and Ki67 index (Supplemental Figure 1B).

### TPX2 nuclear expression and CIN

TPX2 mRNA shows the highest correlation with CIN among all detected RNA transcripts across multiple solid tumors, including primary human breast cancers [25, 27]. We therefore asked if TPX2 nuclear expression as measured by IHC correlated with known drivers of CIN. One mechanism that can bring about spindle assembly defects is centrosome amplification (CA), in which supernumerary centrosomes drive asymmetric cell divisions and cause chromosome segregation errors in mitosis [38–40]. TPX2 is critical for assembly of the mitotic spindle, which ensures the accurate segregation of chromosomes during mitosis. Spindle assembly defects lead to the random loss or gain of whole chromosomes or large portions of chromosomes secondary to defective mitoses, which is the hallmark of CIN. We had previously scored this tumor cohort for CA by performing immunofluorescence for the bona fide centrosome markers pericentrin and polyglutamylated tubulin, and then enumerating centrosome number in a blinded fashion across at least 30 cells in each tumor. We found that tumors with higher TPX2 nuclear expression tended to have a higher incidence of CA, although only the difference between low and high TPX2 nuclear expression was statistically significant (Fig. 2c).

Another mechanism that can facilitate CIN is loss of p53 function and TP53 is among the most mutated genes in breast cancer [41–44]. Thus, we sought to compare TPX2 nuclear expression with TP53 mutational status. Although it was not possible to sequence the TP53 gene in all 253 patient samples, IHC for p53 is a proxy for its mutational status, as most deleterious mutations in TP53 typically cause complete loss or destabilization of p53 protein expression, leading to an “all or nothing” IHC staining pattern. Because this pattern has not been formally demonstrated to be a proxy for TP53 mutation in breast cancer, we instead refer to these tumors as having “aberrant p53 IHC” [45]. We found that tumors with intermediate or high TPX2 nuclear expression showed a significantly higher rate of aberrant TP53 IHC compared to tumors with low TPX2 nuclear expression (Fig. 2d).

We sought additional evidence that tumors with elevated TPX2 expression demonstrate an increased incidence of p53 mutation, and analyzed the provisional invasive breast carcinoma cohort within the The Cancer Genome Atlas (TCGA) [46, 47]. Within this cohort of 499 tumors with RNAseq datasets, we compared the tumors with pathogenic TP53 mutations to tumors in which TPX2 mRNA expression levels were greater than 1.5 standard deviations above the mean (Supplemental Figure 1C). This demonstrated a highly significant co-occurrence of TP53 mutations with TPX2 mRNA overexpression (*P* < 0.001).

Finally, we sought to determine whether TPX2 nuclear expression correlated with CIN itself. We had previously scored the entire cohort for CIN using the gold standard method of CIN analysis, which involves enumerating centromeric FISH probes for loss or gain of chromosomes across at least 10 nuclei per tumor [29]. This approach also allows for the determination of ploidy. Tumors with intermediate and high TPX2 nuclear expression had an elevated ploidy compared to the low TPX2 nuclear expression group, however post-hoc testing revealed that this correlation is only statistically significant between the low and high TPX2 nuclear expression groups (Fig. 2e). In a univariate analysis, tumors with intermediate and high TPX2 nuclear expression also showed elevated incidence of CIN compared to tumors with low TPX2 nuclear expression (Fig. 2f).

To determine whether the power of TPX2 nuclear expression to predict CIN was independent of relevant clinical and pathologic parameters, two linear regression models were fit. The first model adjusted for TPX2, TP53 mutational status, Ki67, ploidy, average centrosome number per cell, and the age of the patient. Because one of the purposes of our study was to identify a modality to predict CIN independent of measuring ploidy, a second model was also fitted which omitted ploidy. In both models, TPX2 was found to have no significant independent association with CIN (*P* = 0.64 and *P* = 0.89 respectively). A graphical analysis of the residuals suggested no significant departure from model assumptions. However, given that CIN is a continuous proportion naturally bounded by 0 and 1, beta regression was also considered. In both model settings (with and without adjusting for ploidy), TPX2 nuclear expression was again found not to be independently associated with CIN (*P* = 0.49 and *P* = 0.93 respectively).

### TPX2 nuclear expression and survival

**Fig. 3:**
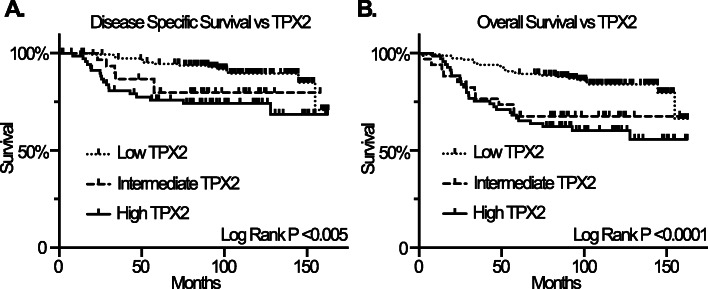
**a** Kaplan-Meier curve showing relationship between disease-specific survival and TPX2 nuclear expression (log rank P < 0.0001). **b**, Kaplan-Meier curve depicting relationship between overall survival TPX2 nuclear expression (log rank *P* < 0.005)

We sought to determine whether TPX2 nuclear expression correlated with disease-specific survival (DSS) and/or overall survival (OS) using log-rank (Mantel-Cox) testing. This revealed that TPX2 nuclear expression significantly correlates with both DSS (*P* < 0.005) and OS (*P* < 0.0001), with lower levels of TPX2 expression correlating with improved survival (Fig. 3a and b). However, a Cox proportional hazards model adjusted for age of diagnosis and Ki67 showed that the predictive value of TPX2 on survival is not independent of Ki67 (*P* = 0.789). Moreover, at least some of the relationship between TPX2 and survival is reflected by the enrichment of triple negative breast cancers within the TPX2 high group (Supplemental Figure 1D and Supplemental Table 1). As expected, when compared to ER+ or HER2+ breast cancers within our cohort, the triple negative breast cancers displayed a higher pathologic grade, higher clinical stage, higher frequency of CIN, higher frequency of aberrant TP53 IHC, higher Ki67 index, higher average ploidy, and higher average centrosome number (Supplemental Tables 1 and 2). Not surprisingly, they also showed worse DSS and OS (Supplemental Figure 1E and F).

## Discussion

Numerous studies have identified TPX2 RNA levels as broadly predictive of aggressive behavior, reduced response to therapy, and poor survival across multiple solid malignancies, including breast cancer [16–22]. Additionally, depletion of TPX2 can suppress proliferation and promote apoptosis [17]. We and others have shown that TPX2 mRNA transcripts are the most predictive of CIN in breast cancers and other solid malignancies [25, 27]. In this study, we sought to compare TPX2 protein expression levels to a broad set of clinicopathologic parameters in a large patient-derived cohort of primary breast cancers. Our findings demonstrate that TPX2 nuclear expression broadly correlates with markers of aggressive behavior, genetic instability, and poor clinical outcome.

Given that the most studied role for TPX2 is in mitotic spindle assembly, a process that begins outside of the nucleus prior to nuclear envelope breakdown, we were surprised that there was very little variability in cytoplasmic TPX2 levels within our cohort and that TPX2 cytoplasmic expression levels did not correlate with any clinicopathologic parameters. However, it should be noted that the spindle assembly function of TPX2 takes place only during the brief period of mitosis, and that only a small minority of tumor cells will be in mitosis at any given time. Thus, we were not able to specifically evaluate TPX2 levels in mitotic cells. On the other hand, we were also surprised by the wide variability in nuclear TPX2 expression across our cohort and how strongly this correlated with markers of tumor aggression and poor outcome. The reasons for this are not immediately clear, as the roles of nuclear TPX2 are only now being elucidated. However, the recent discovery that TPX2 plays a critical role in resolving stalled DNA replication forks that occur in the setting of DNA damage provides a potential explanation [3]. Loss of TPX2 in this context compromises DNA end resection, BRCA1 recruitment, and homologous recombination, and triggers replication fork instability. Thus, increased TPX2 nuclear expression may enable a more robust response to DNA damage and enable more cells to successfully traverse S-phase. We propose that future studies examine whether low TPX2 nuclear expression is predictive of radiation response in breast cancer.

We find that TPX2 nuclear expression is significantly associated with several mechanisms implicated in the genesis of CIN. One mechanism that can promote CIN is supernumerary centrosomes, and our findings indicate that cells with high nuclear TPX2 have a greater number of centrosomes per cell. This is interesting, as TPX2 is known to assist in centrosome clustering and thus its increased expression could enable cell survival in the presence of centrosome amplification [48]. We also analyzed the relationship between p53 mutational status and TPX2 nuclear expression, because loss of p53 is hypothesized to enable the development of CIN [41–43]. Activation of TP53 also triggers transcriptional repression of many cell cycle related genes via the dimerization partner, RB-like, E2F and multi-vulval class B (DREAM) complex, of which TPX2 is a target [49]. Because we lacked the resources to sequence the TP53 gene across all tumors in this large cohort, we instead utilized p53 IHC as a practical but imperfect surrogate. We found that increased TPX2 nuclear expression strongly correlated with an aberrant p53 IHC pattern that supports TP53 mutation in these tumors. This finding was supported by data from the TCGA, in which tumors with elevated TPX2 RNA showed a similar correlation with TP53 mutation. It is unclear why TPX2 RNA levels in the TCGA correlated with nuclear and not cytoplasmic TPX2 protein levels by IHC. However, these findings suggest that TP53 mutation may lead to DREAM complex misregulation and increased TPX2 expression. Together with the centrosome amplification data, we hypothesize that early TP53 mutation may lead to an upregulation in TPX2 levels, which ultimately correlates with genomic instability. Alternatively, overexpression of TPX2 may reduce mitotic fidelity, and further cellular proliferation in this setting is facilitated by loss of p53 function.

Finally, we find that while TPX2 nuclear expression correlates with CIN in univariate analyses, it is not independently predictive of CIN when Ki67, TP53 mutation, average centrosome number, patient age, and/or ploidy are included in the analysis. It is thus unlikely that TPX2 IHC will have practical utility in identifying tumors with CIN. The strong TPX2 correlation with Ki67 suggests that higher TPX2 nuclear expression could instead be a generic marker of higher proliferative rate, which is itself associated with CIN. Given the increasing prevalence of next-generation sequencing (NGS) data associated with newly-diagnosed patient tumors, we believe that future studies should focus on elucidating CIN status from NGS datasets.

An additional limitation of this study is that it does not assess directness of the observed correlations. Nevertheless, this is a large study directly comparing well-localized TPX2 expression with outcomes and clinicopathologic parameters in a well-characterized cohort of breast cancer subjects with long-term follow up.

## Conclusions

Increased TPX2 nuclear expression identified by IHC can enable the identification of breast cancers with poor clinical behavior, elevated proliferative rate, high incidence of pathologic TP53 mutations, and greater genomic complexity. However, it is not independently predictive of CIN. The higher rate of proliferation and genomic instability in tumors with high TPX2 nuclear expression may explain why this marker is associated with poor clinical behavior in this and other studies.

## Supplementary Information

**Additional file 1: Supplemental Table 1** Statistical relationships between breast cancer receptor subtype and age, tumor grade, clinical stage, tumor histology, presence or absence of lymph node metastases at the time of diagnosis, TP53 IHC results, chromosomal instability (CIN), and TPX2 IHC results. Percentages are rounded to the nearest whole number and may not sum to 100%. **Supplemental Table 2** Statistical relationships between breast cancer receptor subtype and tumor size, Ki67 index, ploidy, and centrosome number.

**Additional file 2: Supplemental Fig. 1 A**, Correlation between TPX2 RNA and TPX2 protein expression in 105 primary human breast cancers derived from The Cancer Genome Atlas (solid line = best fit, dashed lines = 95% CI). **B,** Correlation between TPX2 nuclear expression and Ki67 index in study cohort (solid line = best fit). **C**, Co-occurrence of pathologic TP53 mutations and TPX2 mRNA overexpression in The Cancer Genome Atlas provisional breast invasive carcinoma cohort (*P* < 0.001). **D,** Percent of estrogen receptor positive (ER+), human epidermal growth factor receptor 2 positive (HER2+), and ER negative, progesterone receptor (PR) negative, and HER2 negative (triple negative) tumors within the TPX2 low, intermediate, and high groups. **E,** Kaplan-Meier curve showing relationship between disease-specific survival and receptor status (log rank *P* < 0.03). **F**, Kaplan-Meier curve depicting relationship between overall survival and receptor status (log rank P < 0.001).

## Data Availability

TPX2 and p53 immunohistochemical scoring is available from the author upon reasonable request. Linked clinical and pathological information for the utilized TMAs is available from the University of Wisconsin Translational Science Biocore biobank.

## Abbreviations

53BP1: Tumor Protein P53 Binding Protein 1
BRCA1: Breast Cancer 1
CIN: Chromosomal instability
ER: Estrogen receptor
DSS: Disease-specific survival
HER2: Human epidermal growth factor receptor 2
IHC: Immunohistochemistry
OS: Overall survival
PR: Progesterone receptor
RAD51: RAD51 Recombinase
TCGA: The Cancer Genome Atlas
TMA: Tissue microarray
TPX2: Targeting Protein for Xenopus Kinesin Like Protein 2
