# Supplementary material for: High nuclear TPX2 expression correlates with TP53 mutation and poor clinical behavior in a large breast cancer cohort, but is not an independent predictor of chromosomal instability
Source: BMC Cancer. 2021 Feb 23;21:186. doi: 10.1186/s12885-021-07893-7 (PMC7901195; doi:10.1186/s12885-021-07893-7)
Supplement: Supplementary file 1 — Additional file 1: Supplemental Table 1 Statistical relationships between breast cancer receptor subtype and age, tumor grade, clinical stage, tumor histology, presence or absence of lymph node metastases at the time of diagnosis, TP53 IHC results, chromosomal instability (CIN), and TPX2 IHC results. Percentages are rounded to the nearest whole number and may not sum to 100%. Supplemental Table 2 Statistical relationships between breast cancer receptor subtype and tumor size, Ki67 index, ploidy, and centrosome number. [file 12885_2021_7893_MOESM1_ESM.docx]

**Supplemental Table 1.**

|  | **Breast Cancer Subtype** | | |  |
| --- | --- | --- | --- | --- |
| **Parameters** | **ER+**  **(n = 198)**  **n (%)** | **HER2+**  **(n = 32)**  **n (%)** | **Triple Negative**  **(n = 24)**  **n (%)** | 𝛘**2 (P-value**) |
| **Age** |  |  |  |  |
| ≤50 years | 86 (43) | 16 (50) | 7 (29) | 2.5 (0.282) |
| >50 years | 112 (57) | 16 (50) | 17 (71) |  |
| **Tumor Grade** |  |  |  | **59.6 (<0.0001)** |
| 1 | 52 (26) | 3 (9) | 0 (0) |  |
| 2 | 100 (51) | 11 (34) | 1 (4) |  |
| 3 | 45 (23) | 18 (56) | 23 (96) |  |
| **Clinical Stage** |  |  |  | **15.7 (0.0034)** |
| 1 | 94 (47) | 8 (25) | 3 (12) |  |
| 2 | 85 (43) | 18 (56) | 16 (67) |  |
| 3 | 19 (10) | 6 (19) | 5 (21) |  |
| **Histology** |  |  |  |  |
| Ductal | 163 (83) | 30 (94) | 23 (96) | 4.0 (0.0823) |
| Other | 34 (17) | 2 (6) | 1 (4) |  |
| **Regional Lymph Nodes** |  |  |  |  |
| Positive | 81 (41) | 18 (56) | 11 (46) | 2.7 (0.258) |
| Negative | 117 (59) | 14 (44) | 13 (54) |  |
| **TP53** |  |  |  |  |
| Aberrant | 28 (17) | 12 (40) | 18 (75) | **48.3 (<0.0001)** |
| Wild Type | 164 (83) | 18 (60) | 6 (25) |  |
| **CIN** |  |  |  |  |
| Present | 100 (51) | 25 (78) | 12 (50) | **8.6 (0.0134)** |
| Absent | 98 (49) | 7 (22) | 12 (50) |  |
| **TPX2** |  |  |  | **46.3 (<0.0001)** |
| Low | 141 (72) | 17 (53) | 2 (8) |  |
| Intermediate | 23 (12) | 6 (19) | 4 (17) |  |
| High | 33 (17) | 9 (28) | 18 (75) |  |

**Supplemental Table 2.**

|  | **Breast Cancer Subtype** | | |  |
| --- | --- | --- | --- | --- |
| **Parameters (average)** | **ER+**  **(n = 198)**  **mean** | **HER2+**  **(n = 32)**  **mean** | **Triple Negative**  **(n = 24)**  **mean** | **ANOVA**  **F (P-value**) |
| **Tumor Size (cm)** | 23.07 | 27.44 | 31.33 | **3.37 (0.0359)** |
| **Ki67 Index (%)** | 14.47 | 22.13 | 52.75 | **43.7 (<0.0001)** |
| **Ploidy (N)** | 2.23 | 2.62 | 2.49 | **9.3 (<0.0001)** |
| **Centrosome Number/Cell** | 1.83 | 1.91 | 2.29 | **3.6 (0.0299)** |
