# Supplementary figures and images for: High nuclear TPX2 expression correlates with TP53 mutation and poor clinical behavior in a large breast cancer cohort, but is not an independent predictor of chromosomal instability
Source: BMC Cancer. 2021 Feb 23;21:186. doi: 10.1186/s12885-021-07893-7 (PMC7901195; doi:10.1186/s12885-021-07893-7)

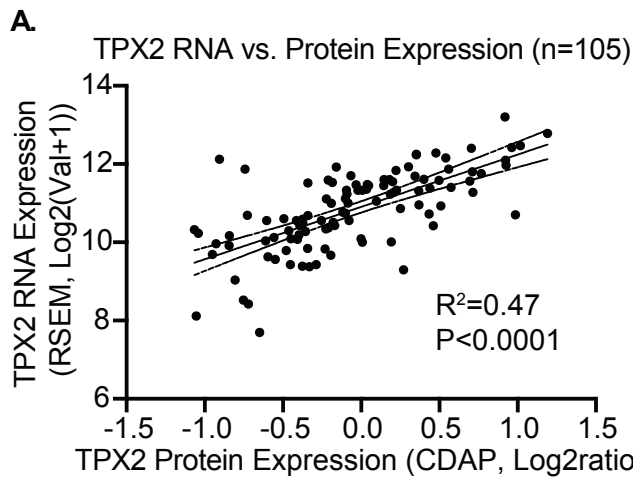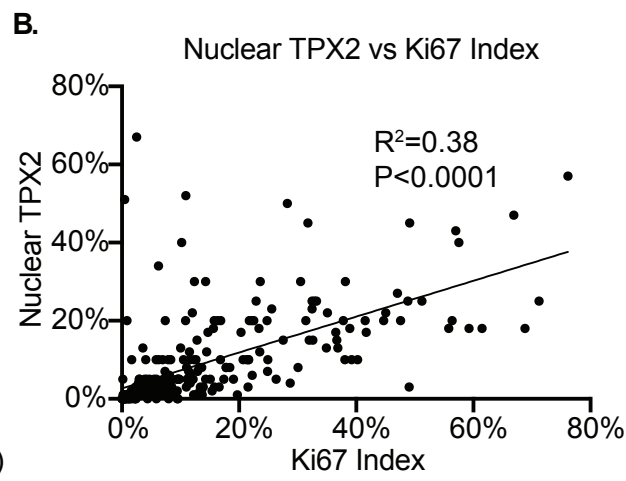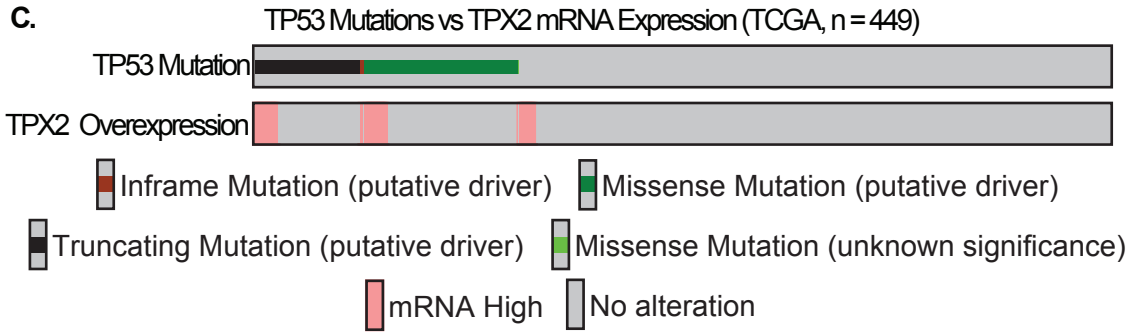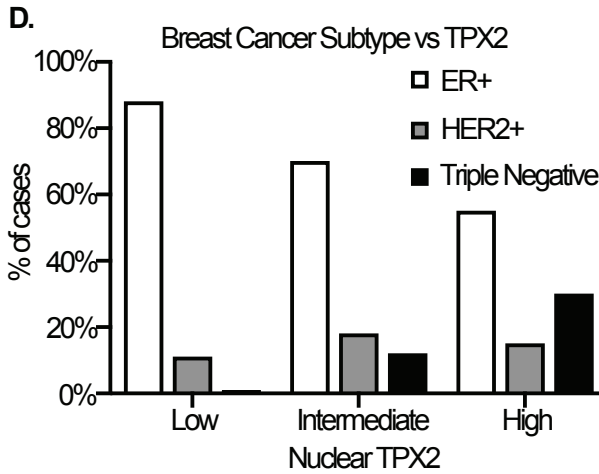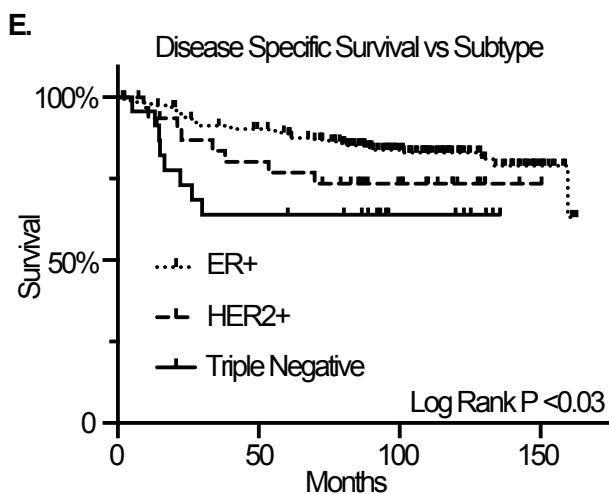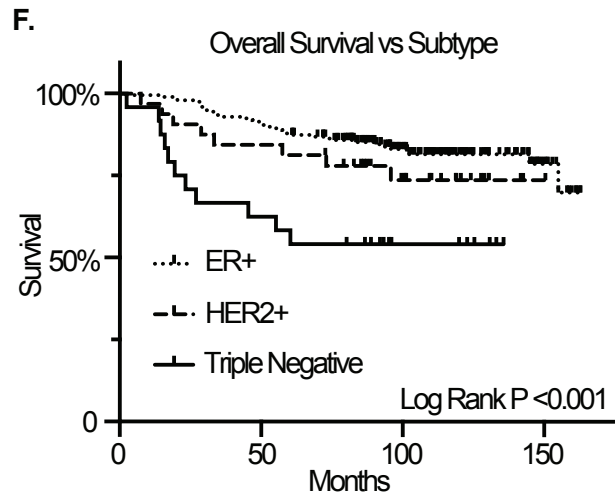

Supplement: Supplementary file 2 — Additional file 2: Supplemental Fig. 1 A, Correlation between TPX2 RNA and TPX2 protein expression in 105 primary human breast cancers derived from The Cancer Genome Atlas (solid line = best fit, dashed lines = 95% CI). B, Correlation between TPX2 nuclear expression and Ki67 index in study cohort (solid line = best fit). C, Co-occurrence of pathologic TP53 mutations and TPX2 mRNA overexpression in The Cancer Genome Atlas provisional breast invasive carcinoma cohort (P < 0.001). D, Percent of estrogen receptor positive (ER+), human epidermal growth factor receptor 2 positive (HER2+), and ER negative, progesterone receptor (PR) negative, and HER2 negative (triple negative) tumors within the TPX2 low, intermediate, and high groups. E, Kaplan-Meier curve showing relationship between disease-specific survival and receptor status (log rank P < 0.03). F, Kaplan-Meier curve depicting relationship between overall survival and receptor status (log rank P < 0.001). [file 12885_2021_7893_MOESM2_ESM.pdf]
